# The risk, perceived and actual, of developing type 2 diabetes mellitus for mothers of preschool children in urban China

**DOI:** 10.1371/journal.pone.0222839

**Published:** 2019-09-27

**Authors:** Jia Guo, Yujia Tang, Honghui Zhang, Lisa Lommel, Jyu-Lin Chen

**Affiliations:** 1 Xiangya School of Nursing, Central South University, Changsha, Hunan, PR China; 2 Department of Hepatobiliary Surgery, Hunan General Hospital, Changsha, Hunan, PR China; 3 School of Nursing, University of California, San Francisco, CA, United States of America; Anglia Ruskin University, UNITED KINGDOM

## Abstract

**Background:**

The perceived risk of developing type 2 diabetes mellitus (T2DM) can motivate individuals to adopt preventive health behaviors. Compared with fathers, mothers of young children often experience unique risk factors for developing T2DM: pregnancy-related weight gain, lifestyle changes related to child care, and the increased incidence of gestational diabetes mellitus. Understanding how actual risk factors affect the perceived risk of developing T2DM can foster effective diabetes prevention interventions for this population. The aims of this study were to describe the risk, perceived and actual, of developing T2DM and to explore the influencing factors of perceived risk for Chinese mothers of preschool children in China.

**Methods:**

A multisite, cross-sectional survey was conducted and included 176 mothers (mean age of 31.19 years old) of preschool children (aged 3–7 years old) from four preschools in Changsha, the capital city of Hunan Province, China. The overall perceived risk of developing T2DM was measured by one item “Your own personal health risk is at almost no risk, slight risk, moderate risk or high risk from diabetes” from the Risk Perception Survey for Developing Diabetes (RPS-DD). PRS-DD and the Chinese version of the Canadian Diabetes Risk Assessment Questionnaire (CHINARISK) were used to assess perceived risk related worry, personal control, optimistic bias, and diabetes risk knowledge and actual risk of T2DM. Mothers also reported their height, weight, and waist circumference followed by the NIH protocol. Pearson correlation and stepwise multivariate linear regression were used to explore how the actual risk factors affected the perceived risk of developing diabetes (RPS-DD)).

**Results:**

Nearly 90% of mothers perceived almost no/slight risk for developing diabetes. Nearly half of the mothers had parents or siblings with diabetes. Roughly 70% of the mothers did not eat five servings of fruits and vegetables per day, and more than 50% did not exercise at least 30 minutes a day. In the five stepwise multivariate linear regression models, young mothers (95% CI .400–1.311) and those with a family history of diabetes (95% CI -0.74- .000) were founded a higher overall perceived risk. Mothers who reported more sedentary time (95% CI -0.029- -0.008) and less physical activity had less personal control (95% CI -0.354- -0.046). Mothers with more sedentary time had more worries about developing T2DM(95% CI 0.008–0.035) . Mothers who were older (95% CI -0.440–0.055) or had more physical activities (95% CI 0.003–0.048) had more optimistic bias of not developing T2DM. Mothers who had a higher education level (95% CI .354–1.422) and a family history of diabetes (95% CI .029–2.231) had more diabetes risk knowledge of developing T2DM.

**Conclusion:**

This study found that Chinese mothers of preschool children in urban areas reported low perceived risk of developing T2DM, although they have actual risk factors. These women did not associate anthropometric, health history, or health behavior factors with the risk of developing T2DM. Anthropometrics and risk factors associated with behavioral risk factors may be the focus of diabetes prevention programs.

## Introduction

China has the largest number of people with undiagnosed diabetes worldwide (roughly 61.3 million) or 53.6% of its population [1]. In a 7-year, nationwide, prospective study of 512, 869 adults in China, diagnosed diabetes was found to be more common in urban than rural areas, 8.1% versus 4.1%, respectively [2]. Ninety-five percent of people with diabetes have type 2 diabetes mellitus (T2DM). The early identification of the risk factors of developing T2DM among undiagnosed populations could improve early diagnosis and prevention.

Sociodemographic factors (e.g., age, gender, ethnicity, education), personal health history (e.g., hypertension, history of gestational diabetes, family history of diabetes), and urbanization-related lifestyle changes (e.g., unhealthy diet, increased sedentary time) are found to be risk factors for developing T2DM [3–5]. Compared with fathers, mothers of young children face additional pregnancy-related risk factors for developing T2DM including weight gain, gestational diabetes mellitus, and delivering a baby over 4 Kg [6]. However, few articles in the literature have reported on the actual risk of developing T2DM for women with young children in China, although the risk could be evaluated by noninvasive screening tools, which are widely used in Western countries [1].

Perceived risk is defined as “perceived probability, likelihood, or susceptibility to harm” [7]. Several health behavior models indicate that perceived risk is a critical determinant of appropriate risk-reduction behavior [8,9]. If high-risk individuals do not believe that they are at increased risk, they may not respond to efforts that promote risk-reducing behaviors (e.g., engaging in physical activity and eating a healthy diet) or screening [10]. Low perceived risk of developing diabetes can be a potential barrier to diabetes prevention [11]. Understanding the factors that influence the perceived risk of developing T2DM will provide the evidence necessary to develop diabetes prevention programs, especially for mothers of young children.

Previous studies have found that undiagnosed populations, even those at high risk, did not necessarily perceive themselves to be at increased risk [11,12]. For example, more than two thirds of an overweight population aged 45 years and over in the United States (US) had few concerns about getting diabetes in the future [12]. In another example, one third to one half of well-educated women in Australia, Canada, and the US with a recent history of gestational diabetes perceived themselves to be at low risk of developing diabetes [11,13,14]. In developing countries like China, there is a lack of evidence on the perceived risk of developing T2DM among women.

Chinese mothers of young children may face unique, lifestyle-related risk factors for developing T2DM due to pregnancy or the transition to motherhood. According to Chinese custom, women are encouraged during pregnancy and the breast feeding period to adopt a high-calorie diet that includes plenty of eggs or meat, chicken soup, and brown sugar water [15]. They are also taught to avoid eating raw and cold food such as fruits and vegetables during the breast feeding period [16,17]. Additionally, mothers were reported to be too busy with taking care of the family and lack of time to exercise [18]. It is important to understand their risk of developing T2DM based on their lifestyle and physical characteristics (such as weight status) and their perceived risk of getting this disease.

The purposes of the study included threefold: (1) to describe the perceived risk of T2DM for mothers of preschool children in China; (2) to identify the actual risk of developing T2DM for mothers of preschool children; and (3) to explore the factors associated with the perceived T2DM risk for mothers of preschool children.

## Materials and methods

### Sampling

A multisite, cross-sectional study design was used. Participants in Changsha, Hunan Province, China, were recruited through four local preschools, which included two public and two private preschools. A self-report questionnaire was used to measure perceived risk and the actual risk factors for developing T2DM. Mothers were instructed to complete the questionnaires and return them in sealed envelopes to the preschools.

A description of the data collection procedure and the inclusion and exclusion criteria have been reported elsewhere [19]. Briefly, mothers were included in this study if they had at least one child aged 3–5 years old and/or child who could attend preschool activities. Mothers with a history of type 1 or type 2 diabetes were excluded from the study. Potential participants received a package containing information related to the study aims and research consent form. Interested participants were asked to return the signed consent form within 2 weeks in order to participate. Children whose mothers agreed to take part in the study provided verbal consents. Data were collected from October 2015 to January 2016. This study was approved by the Human Research Committee at the University of California, San Francisco, USA, (IRB #15–17239) and Central South University in China (IRB #2015039).

### Assessments

#### Sociodemographic and anthropometric data (the independent variables)

A self-designed, sociodemographic form was used to collect age, education level, occupation, and ethnicity. The waist circumference was measured at the highest point of the iliac crest by mothers following the National Institute of Health in the U.S protocol. Mothers were also instructed to report weight and height and body mass index (BMI; kg/m^2^) was calculated based on self-report data.

#### Actual risk factors of developing diabetes (the independent variables)

The Chinese version of the Canadian Diabetes Risk Assessment Questionnaire (CHINARISK) [20], whose psychometric properties have been verified in Chinese populations, provided a list of actual risk factors. CHINARISK comprises 14 items: 1) age; 2) gender; 3) BMI; 4) waist circumference; 5) Do you usually do some physical activity such as brisk walking for at least 30 minutes each day? (At work/At home); 6) Do you eat 5 servings of vegetables or fruit a day; 7) Have you ever been told by a doctor or nurse that your blood pressure is high; 8) Have you ever taken antihypertensive drugs; 9) Have you ever been found to have a high blood sugar and how did you find it; 10) Have you ever given birth to a large baby weighing 4kg or more; 11) Have any of your blood relatives ever been diagnosed with diabetes; 12) parents’ ethnicity; 13) education; and 14) residence (rural or urban).

Total possible scores range from 0 to 88; higher scores indicate a greater risk of developing diabetes within 10 years. Among four items of the CHINARISK, the response of both ‘no’ and ‘don’t know’ are scored zero. Thus, the two categories of each item were merged according to the scoring rule when we described the score distribution of each item. The cutoff point of CHINARISK for high risk is 30. The anthropometric data measured and reported by the mothers were used to complete the CHINARISK. To assess sedentary time, we included one item from the Family Eating and Activity Habits Questionnaire [21], which queried participants on time watching TV and/or playing computer games after work or school each day.

#### Perceived risk of developing diabetes (the dependent variables)

The overall perceived risk of developing T2DM, perceived risk related worry, personal control, optimistic bias, and Diabetes Risk Knowledge were the dependent variables in this study, which were measured by the Risk Perception Survey for Developing Diabetes (RPS-DD) [22]. Using Brislin’s (1986) translation model for cross-cultural research, our research team translated the survey from English to Chinese. This entailed forward translation, back translation, translation equivalence evaluation, and several translation strategies. Cronbach’s alpha of the RPS-DD in this sample was 0.88.

The overall perceived risk for developing T2DM was assessed by the item “Your own personal health risk is at almost no risk, slight risk, moderate risk or high risk from diabetes” of the Personal Disease Risk subscale of RPS-DD. Details about the mothers’ perceived risk of developing diabetes were captured in responses to the subscales on Worry (item score 1–4; 2 items), Personal Control (item score 1–4; 4 items, Optimistic Bias (item score 1–4; 2 items), and Diabetes Risk Knowledge (item score 0, and 1; 11 items). The Worry, Personal Control, Optimistic Bias subscales were reported as the mean score of the items in each of the subscales. For Diabetes Risk Knowledge subscale, summative score of the correct responses to 11 items was used to assess knowledge level. Higher scores on each subscale indicates more worry of developing diabetes, greater personal control for diabetes risk, more optimistic bias for not developing diabetes, and more knowledge of diabetes risk factors.

### Statistical analysis

Statistical analysis was performed using SPSS (version 24.0; SPSS Inc., Chicago, IL). Data were double-entered and checked for accuracy. The missing data was replaced by mean or medium depending on the distribution. We used descriptive analysis to describe demographic characteristics and all major study variables including perceived and actual risk. Pearson correlation (2-tailed) was used to determine whether an association existed between each of the actual risk factors of developing T2DM and the perceived risk. Eleven of the 14 items of actual risk factors and sedentary time entered the model. Three of the 14 items of actual risk factors (gender, ethnicity and live in urban or rural area) were not selected to enter the model because of lack of variance in this study. We used stepwise multivariate linear regression to determine actual risk factors of developing diabetes as predictors of the higher overall perceived risk. The outcome variables were treated as continuous variables. We set the statistical significance level at *p <* 0.05.

Power analysis used G*Power 3 for a two-tailed test of the null hypothesis for a single regression coefficient in a multiple regression found with sample of 176, 12 predictors and alpha = .05/5 = .01 (for 5 regressions), an effect size is between 0.099 and 0.188 with the power ranges from .965 to .999 [23]. Effect size is calculated from the R^2^ [24].

## Results

### Sociodemographic and anthropometrics characteristics of participants’ families

We approached 250 mothers, 240 (96.0%) of them agreed to participate. 222 mothers (88.8%) returned the questionnaire, 46 mothers (18.4%) who missed over 25% of questionnaire items of RPS-DD were excluded from the analysis. One hundred seventy-six mothers completed the questionnaires. The response rate was 70.4%.

The mean age of preschool children was 3.66 years (*SD* = 0.82); 5 1.1% were girls. The mothers’ mean (*SD*) age was 31.19 years old (4.27); the fathers’ mean (*SD*) age was 33.33 years old (4.51). Roughly 59.7% of the mothers had a college or higher level of education, which was slightly higher than that of the fathers. More fathers had full-time jobs than the mothers, 68.2% vs. 49.4%, respectively. Ethnically, all of the mothers and fathers were Han Chinese.

The mothers’ mean BMI was 19.63 kg/m^2^ (*SD* = 2.77); the fathers’ was 25.55 kg/m^2^ (*SD* = 3.50). Only 5.7% of mothers but nearly two thirds of fathers (66.5%) had a BMI greater than 24 kg/m^2^. The mothers’ mean waist circumference was 76.51 cm (*SD* = 8.04); nearly a quarter of them (23.9%) had a waist circumference greater than 80 cm. The fathers’ mean waist circumference was 91.40 cm (*SD* = 10.09); 71% of them had a waist circumference greater than 90 cm. See Table 1 for complete information on the families’ sociodemographic and anthropometrics characteristics.

**Table 1:** The socio-demographic and anthropometrics characteristics of participated families (N = 176).

Characteristics	Mothers	Fathers	Children
Age, mean (SD), y	31.19 (4.21)	33.33 (4.51)	3.66 (.82)
Range, y	21–45	21–47	3–7
Gender, N (%)			
Female	176 (100%)		90 (51.1%)
Male		176 (100%)	86 (48.9%)
BMI, mean (SD), kg/m^2^	19.63 (2.77)	25.50 (3.36)	15.79 (1.82)
BMI < 24 kg/m^2^	166 (94.3%)	59 (33.5%)	
24 kg/m^2^≤BMI < 28 kg/m^2^	8 (4.5%)	79 (44.9%)	
BMI≥28 kg/m^2^	2 (1.1%)	38 (21.6%)	
Waist circumference, mean (SD), cm	76.51 (8.04)	91.40 (10.09)	N/A
Less than 80cm (for female) or 90cm (for male)	134 (76.1%)	51 (29.0%)	
80-88cm (for female) or 90-98cm (for male)	25 (14.2%)	90 (51.1%)	
Over 88cm (for female) or 98cm (for male)	17 (9.7%)	35 (19.9%)	
Education			N/A
High school degree or below	71 (40.3%)	68 (38.6%)	
College or university degree or above	105 (59.7%)	100 (56.8%)	
Occupation, N (%)			N/A
Full-time job	87 (49.4%)	120 (68.2%)	
Part-time job or no job	89 (50.6%)	56 (31.8%)	
Birth weight, mean (SD), kg	N/A	N/A	3.29 (0.47)
Range, kg			2.30–5.60

### Perceived risk of developing T2DM

The mean (*SD*) score of the overall perceived risk of developing T2DM was 1.64 (0.80), with a range of 1–4. Nearly two-thirds (59.7%) of the mothers answered with almost no risk. Twenty-nine percent of the mothers answered with slight risk. Nearly ten percent (8.5%) of the mothers answered with moderate risk. Only 2.8% of the mothers answered with high risk.

**Fig 1:**
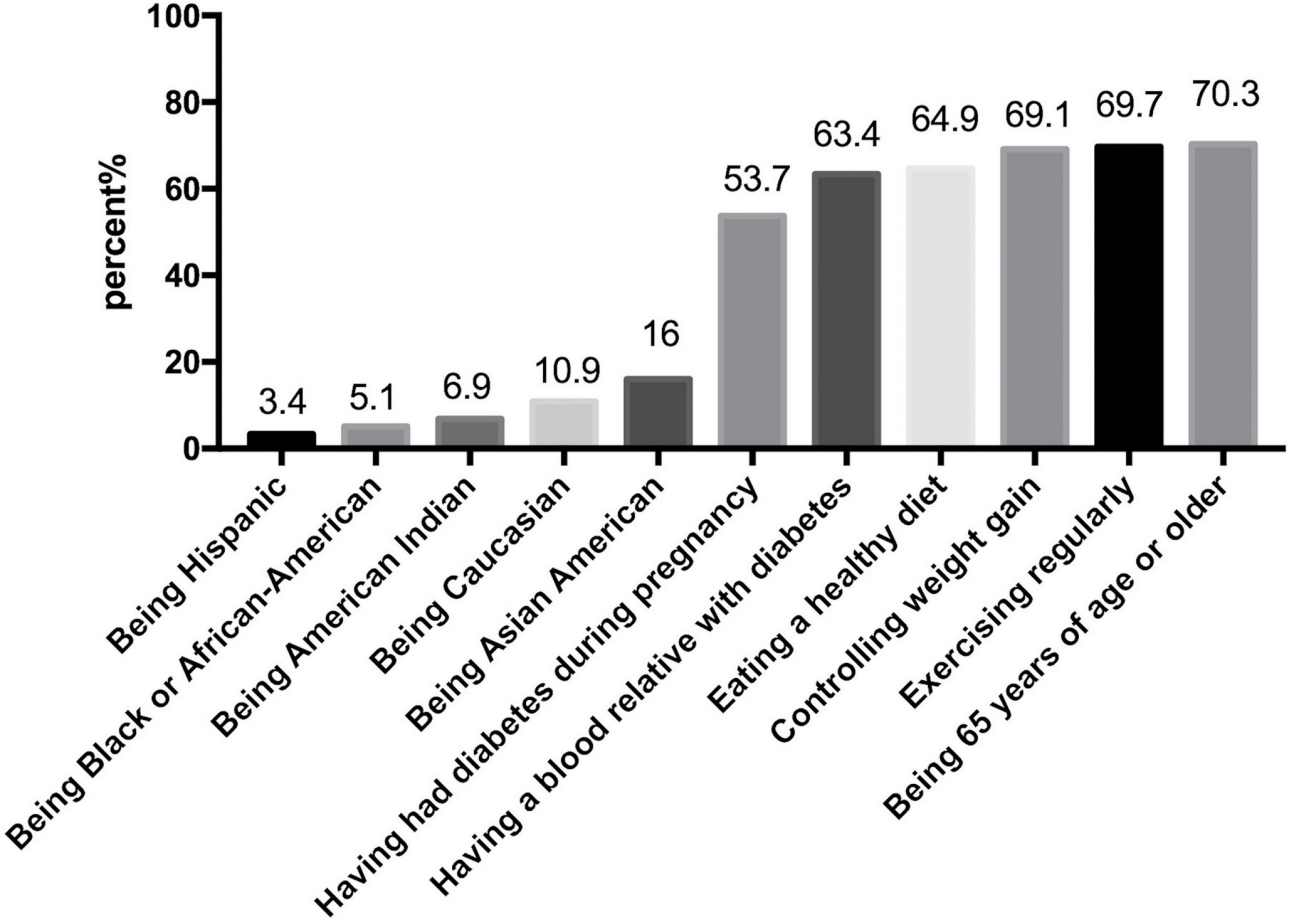
The percentage of the correct answer of diabetes risk knowledge subscale on risk perception of developing diabetes among mothers with preschool children.

Mean (*SD*) scores on the Worry, Personal Control, and Optimistic Bias subscales was 1.99 (0.55), 3.05 (0.40), and 2.91 (0.56), respectively. The mean (*SD*) score on the Diabetes Risk Knowledge subscale was 4.48 (2.23), with a range of 1–11 (Table 2). Two thirds of the mothers knew that getting older or having a family history of diabetes would increase the risk of developing T2DM. Nearly half of the mothers did not know that having gestational diabetes would increase their risk of developing T2DM. A third of the mothers did not know that eating a healthy diet, managing weight within a normal range, or exercising regularly could reduce their risk of developing T2DM. The percentages of the correct answer to each item of Diabetes Risk Knowledge subscale on Risk Perception of Developing Diabetes among mothers with preschool children were displayed in Fig 1.

**Table 2:** The description of the perceived risk of developing T2DM among mothers with preschool children (N = 176).

Perceived risk of T2DM	Mean (SD)	Actual range	Possible range
The overall perceived risk	1.64 (0.80)	1–4	1–4
Worry Subscale	1.99 (0.55)	1–4	1–4
Personal Control Subscale	3.05 (0.40)	2–4	1–4
Optimistic Bias Subscale	2.91 (0.56)	1.5–4	1–4
Diabetes Risk Knowledge subscale	4.48 (2.23)	0–10	0–11

### Actual risk of developing T2DM

The mothers’ mean (*SD*) CHINARISK score was 8.05 (4.99), ranging from 2–32; only one mother had a score greater than the cut-off point of 30. As for health history factors, most of the mothers reported that they did not have hypertension (85.8%); did not take antihypertensive medicine (96.6%); did not have high blood glucose, such as during pregnancy (96.6%); or had not delivered a child weighing 4 kg or more (94.3%). Regarding family history of diabetes, nearly half of the participants (47.7%) reported that their mother, father, sisters, or brothers had diabetes.

As for health behavior factors, less than 50% of mothers reported that they ate more than five servings of fruit and vegetables a day or did physical activities at least 30 minutes a day. Most (92.0%) of the mothers reported sedentary time less than 2 hours a day. The mothers’ mean (*SD*) sedentary time was 0.92 hours (1.06), ranging from 0–7.14 hours. Table 3 lists details of actual risk factors on health history, family history, and health behaviors.

**Table 3:** The actual risk factors of developing T2DM among mothers with preschool children (n = 176).

Health history factors	Categories and scoring	N (%)
Have you ever been told by a doctor or nurse that your blood pressure is high?	No (= 0)	151 (85.8%)
	Yes (= 4)	25 (14.2%)
Have you ever taken antihypertensive drugs?	No/don’t know (= 0)	164 (93.2%)
	Yes (= 4)	12 (6.8%)
Have you ever been found to have a high blood sugar and how did you find it?	No/don’t know (= 0)	170 (96.6%)
	From a blood test /During an illness /During pregnancy (gestational diabetes) (= 14)	6 (3.4%)
Have you ever given birth to a large baby weighing 4kg or more?	No/don’t know (= 0)	166 (94.3%)
	Yes (= 1)	10 (5.7%)
**Family history factors**		
Have any of your blood relatives ever been diagnosed with diabetes?	Other/Don’t know (= 0)	92 (52.3%)
	Mother/ Father/ Brother(s)/ Sister(s) (= 2)	84 (47.7%)
**Health behaviors factors**		
Do you usually do some physical activity such as brisk walking for at least 30 minutes each day? (At work/At home)	Yes (= 0)	82 (46.6%)
	No (= 2)	94 (53.4%)
Do you eat 5 servings of vegetables or fruit a day?	Yes (= 0)	53 (30.1%)
	No (= 2)	123 (69.9%)

Note: age 35–44 (score 0), age 45–54 (score 7); BMI<24 (score 0), 24.0≦BMI<28 (score 4), 28.0≦BMI<28 (score 9), BMI≧32.0 (score 14); waist circumference <80cm (score 0), 80≦waist circumference≦88 (score 4); waist circumference > 88 (score 6); The Han nationality (score 0), The hui nationality (score 6), Other national minorities (score 1); Primary school or less (score 0), High school degree (score 1), University or college degree or above (score 0); Live in cities or towns (score 2); Live in rural areas (score 0)

### Association between actual risk factors and the perceived risk of developing T2DM

Pearson correlations found that having a family history of diabetes is the only variable associated with a higher overall perceived risk of developing T2DM (r = .328, *p* = 0.000). Fewer education years (r = .226, *p* = 0.003), less physical activity (r = -.190, *p* = 0. 014), not eating five servings of fruit and vegetables a day (r = -.166, *p* = 0.032), and more sedentary time (r = -.167, *p* = 0.031) are associated with less personal control. Fewer education years is related to greater worry of developing T2DM (r = -.185, *p* = 0.016). Older age (r = .157, *p* = 0.046) and more physical activities (r = -.223, *p* = 0.004) are associated with greater optimistic bias of not developing diabetes. Mothers who were older (r = .0196, *p* = 0.010) and had more education (r = .368, *p* = 0.000) are associated with higher knowledge of diabetes risk (Table 4).

**Table 4:** Pearson correlation of actual risk factors and perceived risk of developing T2DM among mothers with preschool children.

Actual risk factors	The overall perceived risk	Personal control	Worry	Optimistic bias	Knowledge
**Socio-demographic factors**					
Age	-.126	.133	-.013	.157*	.196*
Education	-.038	.226**	-.185*	-.034	.368**
**Anthropometric factors**					
BMI	.083	.081	.043	.045	.143
Waist circumference	.013	.100	.118	-.020	.034
**Health history factors**					
Have you ever been told by a doctor or nurse that your blood pressure is high?	.069	.081	.075	.128	.116
Have you ever taken antihypertensive drugs?	-.114	.062	-.110	.119	.019
Have you ever been found to have a high blood sugar and how did you find it?	-.122	.013	-.017	.053	-.107
Have you ever given birth to a large baby weighing 4kg or more?	-.062	.059	.017	.123	.070
**Family history factors**					
Have any of your blood relatives ever been diagnosed with diabetes?	.328**	-.018	.135	-.084	.041
**Health behaviors factors**					
Do you usually do some physical activity such as brisk walking for at least 30 minutes each day? (At work/At home)	.142	-.190*	.115	-.223*	-.077
Do you eat 5 servings of vegetables or fruit a day?	.146 (.074)	-.166*	.052	-.068	-.071
Sedentary time	.102	-.167*	.151	-.110	-.076

* Correlation is significant at the 0.05 level (2-tailed).

** Correlation is significant at the 0.01 level (2-tailed).

### Stepwise multivariate liner regression for perceived risk of developing T2DM

Considering all 12 actual risk factors, selected for the purpose of the present study, results of the stepwise multiple regression analysis with backward elimination, revealed that actual risk factors significant for the overall perceived risk and subscales of RPS-DD (Table 5). For higher perceived overall risk of developing T2DM, significant factors included mothers who were younger (sr^2^ = 0.036, *p* = 0.048) and had a family history of diabetes (sr^2^ = 0.125, *p* = 0.000) (R^2^ = 0.153, *F* = 8.504, *p* < 0.001).

**Table 5:** Stepwise multivariate liner regression summary for perceived risk of developing T2DM among mothers with preschool children.

Outcome	Predictor (s)	R^2^	B	SE	sr^2^	t	P	95%CI
The overall perceived risk	overall	.153					.000	
	Family history		.855	.229	0.125	3.730	.000	[.400, 1.311]
	Age		-.037	.019	0.036	-2.002	.048	[-0.74, .000]
The Personal Control subscale	overall	.158					.000	
	Sedentary time		-.018	.005	.099	-3.437	.001	[-0.029, -0.008]
	Physical activity		-.200	.078	.056	-2.578	.011	[-0.354, -0.046]
The Worry subscale	overall	.090					.002	
	Sedentary time		.021	.007	0.090	3.164	.002	[0.008, 0.035]
The Optimistic Bias subscale	overall	.108					.003	
	Physical activity		-.248	.097	0.058	-2.550	.012	[-0.440, 0.055]
	Age		.026	.011	0.046	2.276	.025	[0.003, 0.048]
Diabetes Risk Knowledge subscale	overall	0.139					.001	
	Education		.888	.269	0.094	3.297	.001	[.354, 1.422]
	Family history		1.130	.555	0.036	2.037	.044	[.029, 2.231]

**Note:** sr^2^ indicated factor contribution to the variance of the outcome variable.

For less personal control about developing T2DM, significant factors included mothers who reported more sedentary time (sr^2^ = 0.099, *p* = 0.001) and less physical activity (sr^2^ = 0.056, *p* = 0.011) (R^2^ = 0.158, *F* = 9.385, *p* = 0.000). For more worries about developing T2DM, significant factors included mothers who reported more sedentary time (sr^2^ = 0.090, *p* = 0.002) (R^2^ = 0.090, *F* = 10.013, *p* = 0.002).

For more optimistic bias of not developing T2DM, significant factors included mothers who were older (sr^2^ = 0.046, *p* = 0.025) and had more physical activities (sr^2^ = 0.058, *p* = 0.012) (R^2^ = 0.108, *F* = 6.072, *p* = 0.003). For more diabetes risk knowledge, significant factors included mothers who had a higher education level (sr^2^ = 0.094, *p* = 0.001) and had a family history of diabetes (sr^2^ = 0.036, *p* = 0.044) (R^2^ = 0.139, *F* = 8.090, *p* = 0.001).

## Discussion

We found that Chinese mothers of preschool children perceived a relatively overall low risk of developing T2DM. However, several actual risk factors related to lifestyle behaviors such as insufficient daily intake of fruit and vegetables and insufficient daily physical activities were identified. We found that a family history of diabetes and younger age are *the* key factors associated with an increased perceived risk of developing T2DM in Chinese mothers of preschool-age children.

Although the mothers in our study were well-educated, their perception of the risk of developing T2DM was low, a finding that is consistent with a large survey of Chinese women aged 18–29 years with prior GDM but were less educated [25], compared with our participants. However, the main limitation of this large survey in China is that there was not any information on the psychometric properties of the measurement of perceived risk of developing T2DM.The different level of perceived risk may vary due to different measure of this concept. Nonetheless similar findings among Caucasian women with prior gestational diabetes (mean age 38.7 years) or Latina women (mean age 35.7 years) were found in the previous studies [11,26]. Moreover, the level of optimism for not having T2DM and personal control in our study were not differ from Caucasian and other populations [26,27]. It is possible that mothers in our study were younger or did not have a history of gestational diabetes, leading them to feel less concerned about developing T2DM, or they may have had limited knowledge about the risk factors associated with developing T2DM, as reported in our study.

Compared with 5.6% of mothers with overweight/obesity, the high prevalence (66.5%) of overweight/obesity reported for fathers is of concern. In the nationwide population-based study in China from 2010 to 2014, the prevalence of overweight/obesity among men was 33.8% according to Chinese criteria (BMI≥24.0 kg/m^2^) [28]. The rationales and risk factors for this particular age group participants who are fathers of preschool-age children are not well understood. Future study may need to explore their risk factors.

We found that Chinese mothers of preschool children had limited knowledge about the risk factors for T2DM, despite being well-educated, which is consistent with previous studies of Michigan pharmacists and people in semi-urban Muscat, Oman [27,29]. Although overweight and obesity are risk factors for developing T2DM, two thirds of the Omani adults failed to recognize the risk [29]. Similarly, a multi-ethnic population in the United Kingdom (UK) did not know that a healthy diet and regular exercise can help to reduce the risk of developing T2DM [30]. Thus, lack of knowledge may contribute to low awareness of T2DM and low perceived risk of T2DM risk among Chinese women.

Although the overall actual risk of developing T2DM was low in Chinese women with preschool-age children, more than half of the mothers lacked an adequate level of physical activity, and two thirds of the mothers reported insufficient intake of fruit and vegetables. These findings are consistent with previous studies that reported that nearly two thirds of women in Australia and approximately a quarter of women without T2DM (aged 21–50 years) in the US reported an insufficient level of physical activity [14,31]. A qualitative study of Australian women (mean age 37 years) has suggested that the responsibility of child care, time limitations, and feeling tired were major constraints on postpartum physical activity [32]. Similarly, a study in the UK showed that 68% of the population at high risk of developing T2DM did not meet the recommendation of eating five servings of fruit and vegetables a day [33]. It is likely that our sample of Chinese women with preschool-age children also found it difficult to be physically active and conscientious about eating the recommended amount of fruit and vegetables.

Our findings suggest that perceived risk is not associated with actual risk including BMI, physical activity, diet, and history of gestational diabetes. This echoes the findings of studies of women with a history of gestational diabetes mellitus including American women and Dutch people [11,31,34] [35]. Perhaps our participants’ lack of knowledge on the risks associated with T2DM and time to focus on their own health explains this finding. Future studies are needed to prospectively explore the relationship between perceived risk of developing T2DM and lifestyle behaviors. [34].

We found that having a family history of T2DM is associated with an increased overall perceived risk of developing T2DM, which is supported by studies in the rural US and Australia [14,29,36]. Individuals with family history of a certain disease may feel vulnerable, which may increase their awareness. Of note, we found that mothers of younger age children tended to perceive a higher risk of developing T2DM, which is consistent with a study of a Dutch population aged 50–75 years [35]. By contrast, Zara and colleagues found that getting older was associated with more perceived risk of T2DM among women with prior gestational diabetes mellitus [37]. Some studies have found no significant relationship between age and the perceived risk of T2DM [14,26]. Thus, more studies are warranted to identify potential factors associated with the perceived risk for T2DM.

We found increased sedentary time to be associated with more worry of developing T2DM and less personal control, a finding that is consistent with a study of Australian office workers aged 35–56 years [38]. Somewhat perplexing, we also found that less physical activities were associated with more personal control and being more optimistic about not developing T2DM. This finding might be explained by the limitation of self-reported physical activities, which are often overestimated, especially among a population with smaller BMI or waist circumference [39]. Wearable equipment is recommended to objectively measure physical activities.

## Limitations

The participants hailed from urban areas in Changsha (Southern China area), thus the generalizability of our findings may be limited. Second, the study’s cross-sectional design limited our ability to examine causal relationships between perceived risk and actual risk of developing T2DM. Despite these limitations, our findings on perceived risk and actual risk of developing T2DM are consistent with those reported elsewhere.

## Implications

The degree of perceived vulnerability to a disease can play an important role in initiating preventative behaviors [8]. This study suggests there is a great need of developing programs to increase the awareness of T2DM risk among mothers with preschool children and healthy lifestyle program to improve health, especially on the modifiable actual risk factors such as eating five servings of vegetables and fruit, and pregnancy related information (GDM history or child’s birth weight). Since the dietary practice/habits vary in region and rural areas in China, future study may need to examine how various dietary habits or ethnic cultures influence the perceived risk.

## Conclusion

Chinese mothers of preschool children in urban areas reported low perceived risk of developing T2DM, although they have several actual risk factors and limited knowledge of diabetes risk factors. Health program may include program aimed to increase awareness of T2DM and improve health lifestyle. Future research should also identify risk factors associated with perceive risk for T2DM in man and develop family-based program to improve the health of family.

## Supporting information

S1 File Dataset. (XLSX)
